# Robot-assisted laparoscopic partial gastrectomy combined with endoscopy for gastrointestinal stromal tumors with intraluminal growth: a report of two cases

**DOI:** 10.1093/jscr/rjac416

**Published:** 2022-11-03

**Authors:** Kazuhiro Okamoto, Yuichiro Kato, Fumihiko Yoneyama, Keiko Kimura, Naoya Yamaguchi, Fumitoshi Mizutani, Kosuke Jikei, Hiroshi Kouno

**Affiliations:** Department of Surgery, Nagoya Ekisaikai Hospital, Nagoya, Aichi, Japan; Department of Surgery, Nagoya Ekisaikai Hospital, Nagoya, Aichi, Japan; Department of Surgery, Nagoya Ekisaikai Hospital, Nagoya, Aichi, Japan; Department of Surgery, Nagoya Ekisaikai Hospital, Nagoya, Aichi, Japan; Department of Surgery, Nagoya Ekisaikai Hospital, Nagoya, Aichi, Japan; Department of Surgery, Nagoya Ekisaikai Hospital, Nagoya, Aichi, Japan; Department of Surgery, Nagoya Ekisaikai Hospital, Nagoya, Aichi, Japan; Department of Surgery, Nagoya Ekisaikai Hospital, Nagoya, Aichi, Japan

## Abstract

In cases of gastrointestinal stromal tumor (GIST) with intraluminal growth, determining the minimal resection line is difficult; however, the combined use of endoscopy can overcome this limitation. We performed robot-assisted partial gastrectomy with endoscopy for two cases of internally developed GISTs located on the posterior wall near the esophagogastric junction (EGJ). We confirmed the tumor location and determined minimal surgical margins using endoscopy. The double bipolar method (DBM), which is performed with Maryland bipolar forceps in the right hand and fenestrated bipolar forceps in the left hand, was used to reduce residual gastric damage and prevent tumor damage. The characteristics of robot-assisted surgery made it easier to precisely perform anastomosis of the upper part of the stomach, as compared with laparoscopic surgery, thus minimizing gastric deformity. Both patients were discharged without postoperative complications. In conclusion, robot-assisted partial gastrectomy using the DBM may represent a viable treatment option for gastric submucosal tumors close to the EGJ.

## INTRODUCTION

Gastrointestinal stromal tumor (GIST) is a common mesenchymal tumor, and the principle of surgical treatment is complete resection with negative margins. Laparoscopic resection of gastric GIST is typically performed, and the surgery does not require wide negative margins. However, gastric GIST is often located at the fornix or upper position of the stomach [1], making it difficult to perform minimally invasive local resection and suturing without causing gastric deformity and stricture.

Recently, robotic surgery has been used to overcome the technical limitations of laparoscopic surgery. Robot-assisted gastrectomy can reportedly facilitate lymph node dissection and complex reconstruction [2–4]. In comparison to laparoscopic forceps, the forceps used in robotic surgery can be manipulated at more angles. We applied the double bipolar method (DBM) using Maryland forceps (Intuitive Surgical Inc.) for gastrectomy [5]. The DBM allows for a more flexible angle of incision compared with using the vessel sealer (Intuitive Surgical Inc.) and harmonic scalpel (Intuitive Surgical Inc.), and minimal resection is possible. Furthermore, the damage to the residual stomach is less than that caused by monopolar forceps because of less thermal damage to the surrounding area. Intragastric surgery, such as laparoscopic and endoscopic cooperative surgery (LECS), avoids total gastrectomy or fenestrated resection, particularly for tumors close to the esophagogastric junction (EGJ) [6]; nevertheless, this technique requires expert endoscopists. To achieve basic proficiency in endoscopic submucosal dissection (ESD), trainees should perform at least 30 cases of gastric ESD under expert supervision [7] and 50–100 cases to become proficient [8].

There are no reports on minimal resection using the DBM while simultaneously viewing the endoscope screen and the robot’s field of view and specifically pointing to the tumor margins with the endoscope [9].

## CASE SERIES

### Case 1

**Figure 1:**
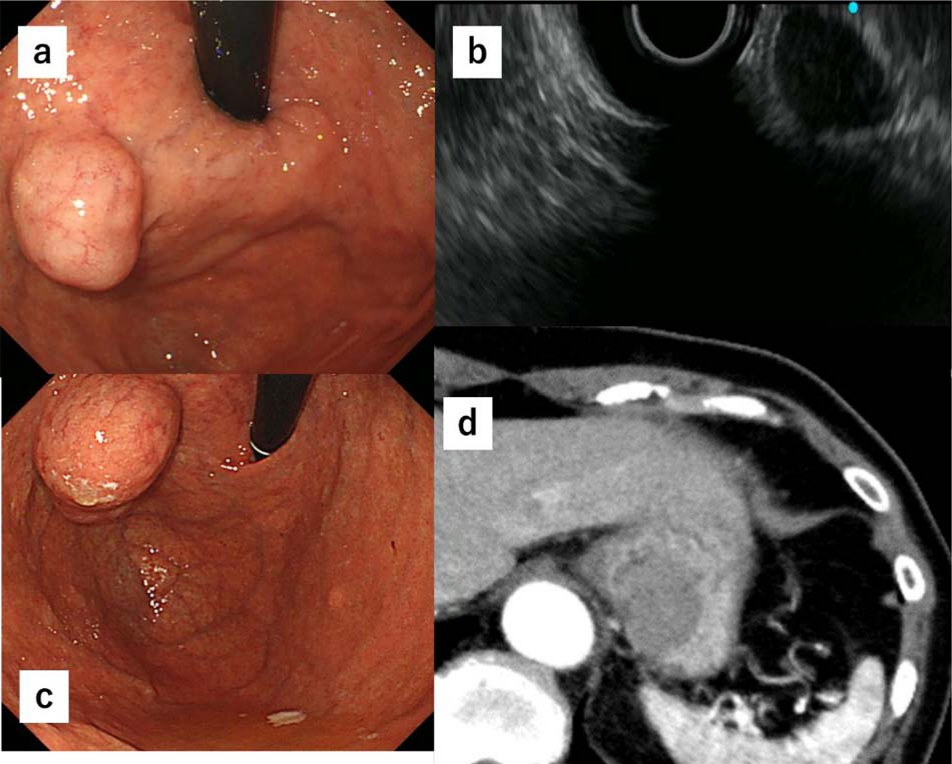
(**a**) A submucosal tumor measuring ∼2 cm is found at the posterior wall of the upper body of the gastric body near the EGJ by upper endoscopy. (**b**) Endoscopic ultrasonography shows a gastric hypoechoic tumor from the fourth layer suggesting GIST. (**c**) The tumor shows increase in size by ∼1 cm compared with 1 year ago. (**d**) CT reveals an intraluminal growth type submucosal tumor measuring 3.5 cm in diameter and no metastasis.

A 74-year-old woman who experienced epigastric discomfort 4 years ago visited our gastroenterology department. Physical examination and laboratory tests revealed no abnormal findings. A submucosal tumor measuring ∼2 cm in diameter was found at the posterior wall of the upper body of the gastric body near the EGJ by upper endoscopy, and endoscopic ultrasonography revealed a gastric hypoechoic tumor from the fourth layer, suggesting GIST (Fig. 1a and b). It was followed up because of its small size. The tumor grew in size gradually, and the patient was referred to our department. The tumor increased in diameter by ∼1 cm over 1 year (Fig. 1c). Computed tomography (CT) revealed an intraluminal growth type submucosal tumor measuring 3.5 cm in diameter and no metastasis (Fig. 1d). After biopsy, the tumor was classified as group 1 (i.e. normal tissue and non-neoplastic lesions).

**Figure 2:**
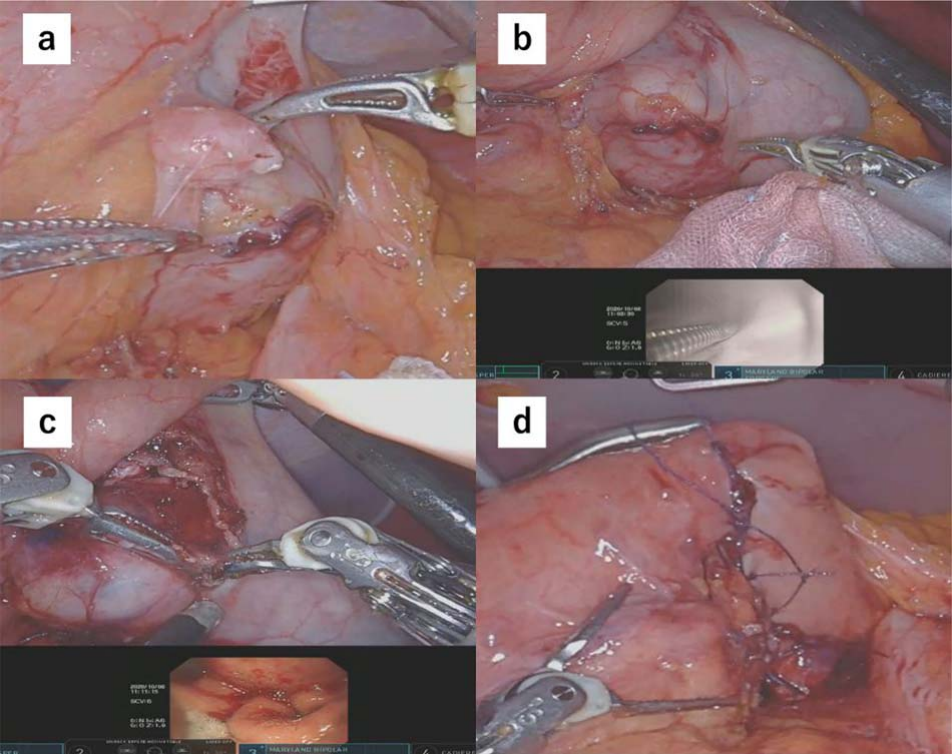
(**a**) After mobilization of the gastric posterior wall, another tumor, which looked like a lymph node, was identified and resected. (**b**) Upper endoscopy shows the tumor and the wedge pushed using biopsy forceps; in this manner, we confirmed the minimum resection line. (**c**) The minimum partial resection was performed using Maryland bipolar forceps according to the line confirmed from inside and outside the stomach. (**d**) Reconstruction was performed using Albert–Lembert sutures.

Due to the tumor location, performing conventional laparoscopic surgery to achieve local resection with sutures that do not damage the EGJ and prevent gastric stricture was difficult. We performed robot-assisted laparoscopic surgery combined with endoscopy to determine the minimum excision margins. Five trocars were placed in the anterior abdomen, guided by a horizontal reference line 8 cm cephalad to the umbilicus. An 8-mm port was inserted into the umbilicus, and another 8-mm port was inserted on the left side of the abdomen. A 12-mm port for use by the assistant was placed between the latter port and the umbilicus. Similarly, an 8-mm port was inserted on the right side of the abdomen, and a 12-mm port was placed between that port and the umbilicus. Our robot-assisted gastrectomy was regularly performed using the DBM, with Maryland- and fenestrated bipolar forceps in the right- and left hand, respectively. We opened the omental bursa, and the anterior wall of the stomach was sufficiently mobilized to confirm the tumor location. After mobilization, another tumor, which looked like a lymph node, was identified and resected (Fig. 2a). Upper endoscopy revealed the tumor, and the wedge was pushed using biopsy forceps; in this manner, we confirmed the minimum resection line (Fig. 2b). The laparoscopic and endoscopic views were displayed on the same monitor so that the surgeon could confirm the position of the biopsy forceps in real time. Minimum partial resection was performed using Maryland bipolar forceps according to the line confirmed from inside and outside the stomach (Fig. 2c). Reconstruction was performed using Albert–Lembert sutures (Fig. 2d). The specimens were retrieved using an endobag, and a drain was placed under the stomach. The operative time was 158 min, and blood loss was 1 mL. The postoperative course revealed no adverse events, and the patient was discharged on postoperative day 6.

**Figure 3:**
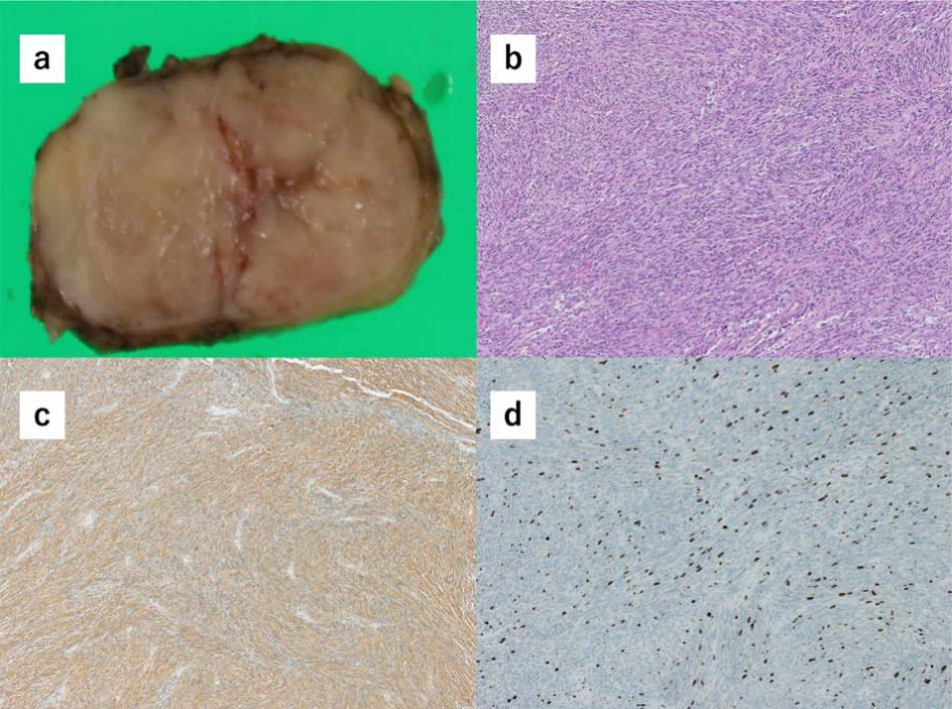
(**a**) Macroscopically, the partial gastrectomy specimen measured 35 mm × 20 mm. (**b**) The histopathology of the specimens reveals that the tumors comprise bundle-like proliferation of spindle-shaped cells and are positive for c-kit. (**c**) The tumors are positive for c-kit. (**d**) The mitotic rate was 3 per 50 HPF, and Ki67 showed a low proliferation rate.

Histopathology of the specimens revealed that the tumors comprised bundle-like proliferation of spindle-shaped cells and were positive for c-kit (Fig. 3a–c). In addition, the tumor-mimicking lymphoma was GIST, which was growing outside the gastric wall. The mitotic rate was 3 per 50 HPF, and Ki67 showed a low proliferation rate (Fig. 3d). These findings indicated that the GISTs were in the low-risk category. There was rare margin involvement (R0).

### Case 2

**Figure 4:**
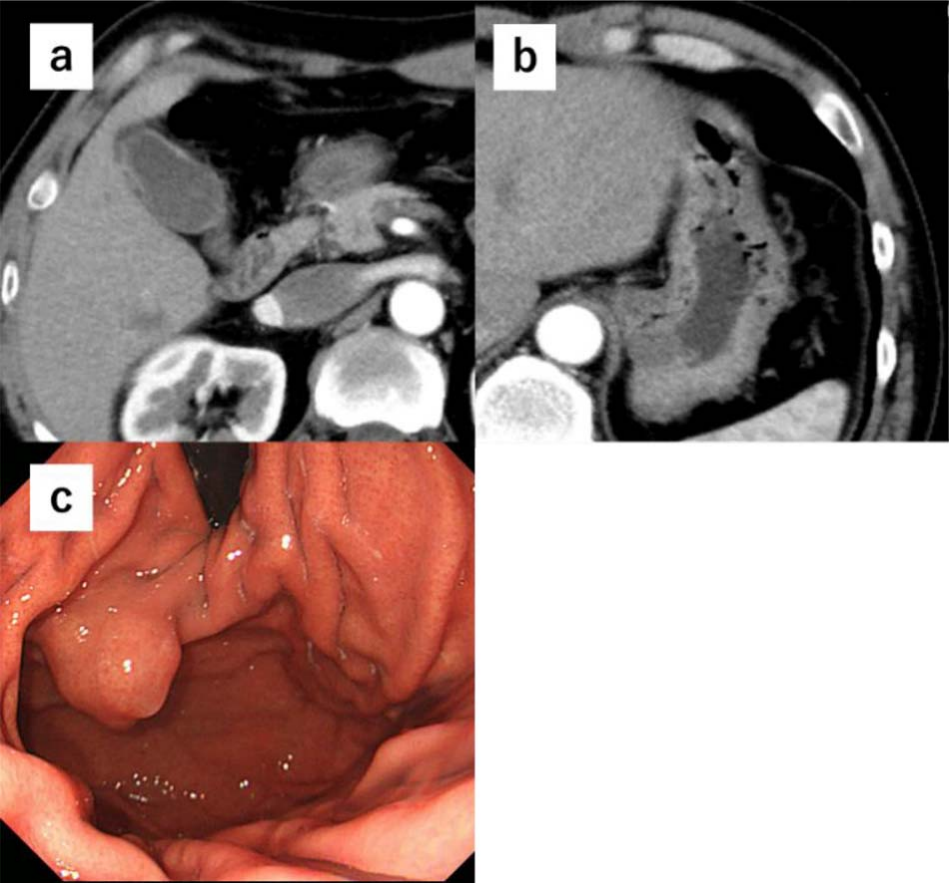
(**a**) CT shows cholecystitis, and no gall bladder is found. (**b**) CT shows cholecystitis, and no gall bladder was found. A submucosal tumor measuring 3.5 cm in diameter was incidentally detected. (**c**) Upper endoscopy reveals the tumor in the anterior wall of the gastric cardia near the EGJ.

**Figure 5:**
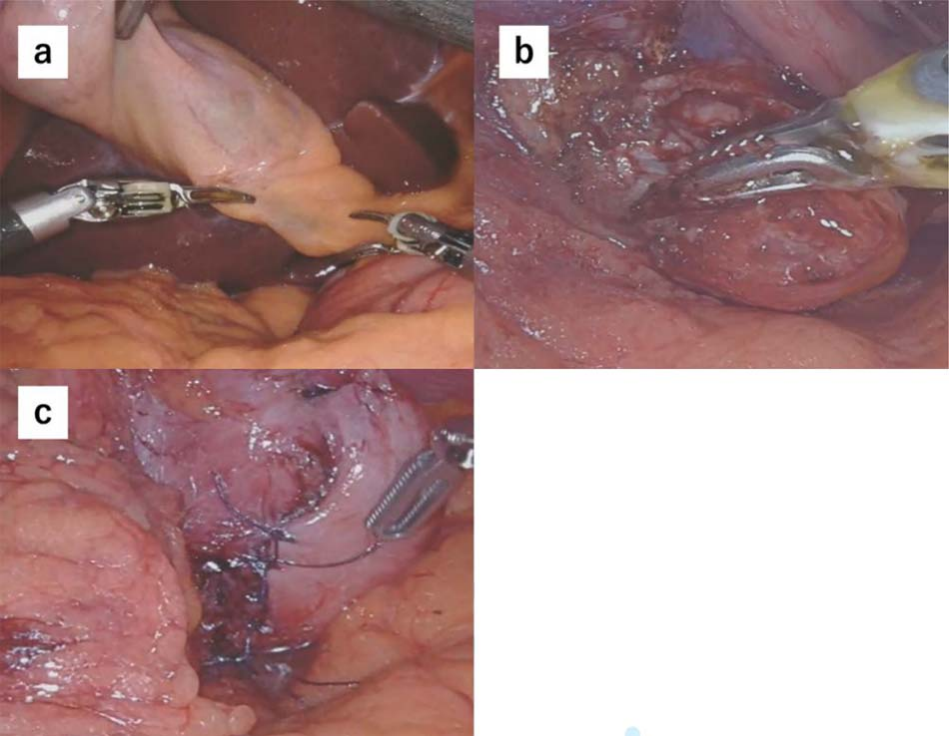
(**a**) We performed robot-assisted cholecystectomy. (**b**) The tumor location was confirmed endoscopically, and the tumor was resected minimally. (**c**) Reconstruction was performed by Albert–Lembert suture.

A 52-year-old man was diagnosed with acute cholecystitis after presenting to the emergency department of our hospital with right upper quadrant abdominal pain. Blood tests revealed the following measurements: white blood cell count, 15 300/mm^3^; C-reactive protein, 0.03 mg/dL; total bilirubin, 1.2 mg/dL; direct bilirubin, 0.7 mg/dL; alkaline phosphatase, 338 U/L; aspartate transaminase, 126 U/L and alanine aminotransferase, 66 U/L. The abovementioned values revealed an inflammatory reaction and mild elevation of hepatobiliary enzymes. CT revealed cholecystitis, and a submucosal tumor measuring 3.5 cm in diameter was incidentally detected (Fig. 4a and b). The patient was treated with antibiotics for cholecystitis and referred to our department for simultaneous surgery. Upper endoscopy revealed a tumor in the anterior wall of the gastric cardia near the EGJ (Fig. 4c). He underwent robot-assisted laparoscopic resection for the tumor, combined with endoscopy and robot-assisted cholecystectomy (Fig. 5a–c). The port positions were the same as those in Case 1. The operative time was 228 min, and blood loss was 1 mL. No adverse postoperative events occurred, and the patient was discharged on postoperative day 7.

**Figure 6:**
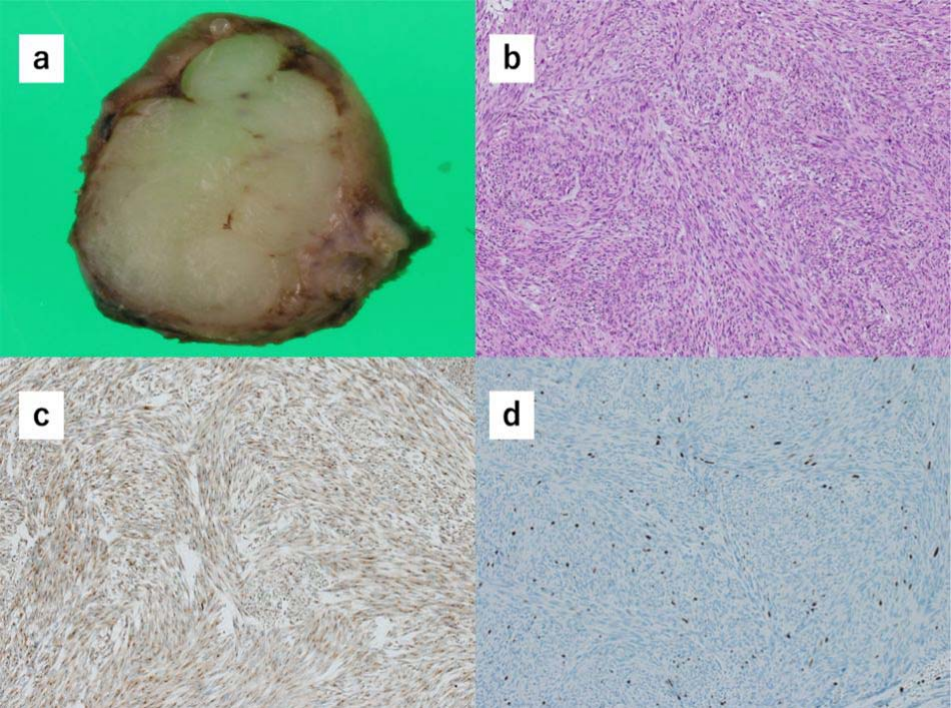
(**a**) Macroscopically, the partial gastrectomy specimen measures 25 mm × 20 mm. (**b**) Histopathology of the specimens reveals that the tumors consist of bundle-like proliferation of spindle-shaped cells and are positive for c-kit. (**c**) The tumors are positive for c-kit. (**d**) The mitotic rate was 7 per 10 HPF, and the Ki67 index rate was 7%.

Similar to Case 1, histopathology of the specimen revealed that the tumors comprised bundle-like proliferation of spindle-shaped cells and were positive for c-kit (Fig. 6a–c). The mitotic rate was 7 per 10 HPF, and the Ki67 index rate was 7% (Fig. 6d). These findings indicated that the GISTs were in the middle-risk category. Rare margin involvement (R0) was noted.

## DISCUSSION

The majority of GISTs occur in the stomach, with an incidence of 50% [10–13]. GIST occurring in the stomach is often located in the fornix and upper side of the stomach [1]. The only curative treatment for GIST is complete resection (R0) with an intact pseudocapsule. Lymph node metastasis is rare, and no prophylactic lymph node dissection is required if metastasis is not diagnosed [14].

For gastric GISTs <2 cm, wedge resection is generally performed by laparoscopic surgery. Moreover, there are reports of partial laparoscopic resection in the literature [15]. Laparoscopic surgery offers the following advantages over open surgery: less pain, earlier food intake, reduced complications and shorter hospital stay [16]. It is difficult to avoid gastrectomy when the tumor is located near the lesser curvature or esophageal junction because wedge resection may cause stricture. However, total gastrectomy or cardiac gastrectomy is considered excessive if postoperative pathology reveals a benign condition. Although a margin of 1–2 cm was traditionally considered necessary for adequate resection, DeMatteo *et al*. showed that tumor size, not the negative microscopic resection margins, determined the survival rate [10]. Therefore, the surgical goal should be complete resection, aiming at negative gross resection margins without routine lymphadenectomy [11, 17].

Robot-assisted surgery has gained tremendous traction among surgeons, as it enables a high-magnification, three-dimensional view in real time and enhanced hand-eye coordination. These advanced systems are used in various reconstructive procedures because of their advantages, allowing delicate movements in small areas during procedures that would otherwise require advanced laparoscopic expertise [18, 19]. Robot-assisted surgery enables easier suturing [20] and reduced gastric deformity and stricture. When laparoscopic surgery is performed in the upper part of the stomach away from the port, suturing accurately and minimally is difficult. In such cases, laparoscopic forceps have limited mobility, especially for tumors in the posterior wall of the upper stomach [10]. Robotic surgery allows for free angulation of the needle, thus minimizing gastric deformity and preventing esophageal stricture.

We usually perform robot-assisted gastric surgery using the DBM, which is executed using Maryland bipolar forceps in the right hand and fenestrated bipolar forceps in the left hand [5]. The advantage of this method is that it is less damaging to the surrounding tissue because the forceps cause less dissipation of current and heat, unlike the harmonic scalpel. Compared with monopolar forceps, thermal damage to the surrounding area is smaller with Maryland bipolar forceps [21], thereby decreasing the damage to the gastric wall during resection. Furthermore, damage to the tumor can be reduced even when the incision is made at the very edge of the tumor. Unlike energy devices such as the vessel sealer and harmonic scalpel, the joint function allows for more angular resection. In contrast, wedge resection using a stapler results in excessive removal of normal gastric tissue. The DBM allows for partial resection without excessive removal of normal tissue and damage to the remaining stomach. Moreover, the ability to grasp the tissue allows for smooth development of the surgical field. This method is particularly useful in gastric surgery, as considerable lifting is required for field development.

In cases of GIST with intraluminal growth, identifying the tumor location outside the stomach is difficult. Even if the tumor location is identified, accurately drawing a minimal line of dissection is difficult. To avoid this, the resection line was confirmed under endoscopic guidance. Because the screen being viewed by the endoscopist could be projected onto the laparoscopic screen at the same time, cooperation between the endoscopist and surgeon was easy. In addition, LECS was indicated, but the tumors were near the EGJ; thus, the expertise of the endoscopist was required. LECS involves endoscopic dissection of the mucosal or submucosal layer with laparoscopic resection of the serosa. The aim of LECS is to efficiently resect benign tumors and preserve as much of the normal stomach as possible [6]. LECS procedures were classified by Min *et al*. according to the location of the gastric submucosal tumor. When wedge resection is difficult on the posterior wall side or near the upper body, especially near the EGJ, inserting laparoscopic forceps into the stomach is recommended (endoscope-assisted laparoscopic transgastric resection, laparoscopic intragastric surgery and single-incision intragastric resection) [22]. In the LECS method, ESD is followed by laparoscopic serosal dissection, which is a precise and time-consuming procedure. However, in the TS-RECS method, the technical advantages of the da Vinci Surgical System (flexible and precise articulated forceps, tremor filtering function, 3D high-definition field of view, etc.) enable each procedure to be performed more efficiently and precisely. Due to the advantages associated with robotic surgery, this technique avoids total gastrectomy or proximal resection for lesions near the EGJ or pylorus while performing R0 resection [23]. However, both procedures require experienced endoscopists for joint endoscopic surgery and cannot therefore be performed at several institutions. To achieve basic proficiency in ESD, trainees should perform at least 30 cases of gastric ESD under expert supervision. Once the endoscopist is able to perform basic techniques, they can progress to ESD, which is performed on tumors located at more difficult sites, such as the upper part of the stomach [7]. Because LECS is not a widely performed procedure, our technique could be performed in various institutions as an extension of their usual surgical procedures.

In conclusion, the postoperative course in either case showed no postgastrectomy syndrome, including small stomach syndrome or congestion symptoms. Robot-assisted gastrectomy with endoscopy is a useful and minimal resection method for gastric GIST that are difficult to locate. Robotic technology will enable more accurate and minimally invasive surgical management. With the development of precision medicine in surgery, surgical methods should be adopted to match the characteristics of individual patients and tumors to derive maximum benefit from the procedure.
